# Nested polymerase chain reaction for amplification of the Cryptosporidium oocyst wall protein gene.

**DOI:** 10.3201/eid0701.700049

**Published:** 2001

**Authors:** S Pedraza-Díaz, C Amar, G L Nichols, J McLauchlin

**Affiliations:** PHLS Central Public Health Laboratory, London, UK.

## Abstract

We developed a sensitive nested polymerase chain reaction procedure for the Cryptosporidium oocyst wall protein (COWP) gene. Amplification and genotyping were successful in 95.2% of 1,680 fecal samples, 77.6% by the unnested and 17.6% by the nested COWP procedure. The COWP gene was amplified from 2,128 fecal samples: 71 from livestock animals and 2,057 from humans. This series included 706 cases from seven drinking water-associated outbreaks and 51 cases from five swimming pool-associated outbreaks, as well as 1,300 sporadic cases.


### Nested Polymerase Chain Reaction for Amplification of the *Cryptosporidium* Oocyst Wall Protein Gene

**Susana Pedraza-Díaz,* Corinne Amar,***
**Gordon L. Nichols,† Jim McLauchlin***
*PHLS Central Public Health Laboratory, London, UK;
†PHLS Communicable Disease Surveillance Centre, London, UK

We developed a sensitive nested polymerase chain reaction procedure for the *Cryptosporidium* oocyst wall protein (COWP) gene. Amplification and genotyping were successful in 95.2% of 1,680 fecal samples, 77.6% by the unnested and 17.6% by the nested COWP procedure. The COWP gene was amplified from 2,128 fecal samples: 71 from livestock animals and 2,057 from humans. This series included 706 cases from seven drinking water-associated outbreaks and 51 cases from five swimming pool-associated outbreaks, as well as 1,300 sporadic cases.

The coccidian parasite *Cryptosporidium* is now recognized as a major cause of waterborne diarrheal disease worldwide (1,2). The exact modes of transmission, however, are often unclear, and the relative importance of foreign travel; consumption of foods, beverages, or water; person-to-person transmission; and infected animals in disease transmission remain to be ascertained (1,2).

Characterization of *Cryptosporidium parvum* by phenotypic and genotypic methods (3-9) has identified two major types associated with human infection: one exclusively from humans and a single nonhuman primate (genotype 1 or human type) and a second in livestock as well as humans (genotype 2 or calf type). Experimental infection of both calves and mice was successful with genotype 2 but not with genotype 1 (4). Polymorphic genes that identify these genotypes include the *Cryptosporidium* oocyst wall protein (COWP) gene (5), the thrombospondin-related adhesive proteins *Cryptosporidium*-1 (TRAP-C1 [6]), and *Cryptosporidium*-2 (TRAP-C2 [4]). These observations concerning the two genotypes of *C. parvum* may reflect the epidemiology of two parasites with distinct and exclusive transmission cycles (4,9) and may represent two species (8).

Address for correspondence: J. McLauchlin, Food Safety Microbiology Laboratory, PHLS Central Public Health Laboratory, 61 Colindale Avenue, London, NW9 5HT, UK; fax: +44 20 8200 8264; e-mail: jmclauchlin@phls.nhs.uk.

We have described the identification by polymerase chain reaction-restriction fragment length polymorphism (PCR-RFLP) of a single human isolate with an unusual COWP genotype, designated genotype 3 (10). Several *Cryptosporidium* species are associated with human disease, including *C. felis*, *C. meleagridis*, and an as-yet-unnamed species designated the dog type (11,12). DNA sequencing of multiple genes from six human isolates of COWP genotype 3 indicates that separate species status is justified; its 18S rDNA sequences are identical to those of *C. meleagridis* (13). Since the host range of the various *Cryptosporidium* species and *C. parvum* genotypes infectious to humans differs, their epidemiology is also likely to differ.

We have described a simple DNA extraction method from whole feces, suitable for amplification of *Cryptosporidium* DNA, and have applied it to 397 cryptosporidiosis cases, including sporadic human and animal cases as well as cases from two large waterborne outbreaks (8,10). In 218 sporadic human cases, DNA could not be amplified from 9% of samples for genotyping by PCR-RFLP analysis of the COWP gene (5,8), despite amplification of 18S rDNA fragments or detection of oocysts by microscopy. The purposes of this study were to develop sensitive methods for identifying *Cryptosporidium* genotypes in DNA extracted from whole feces and to assess the application of these techniques to large numbers of samples.

### Materials and Methods

### Fecal Samples, Oocyst Disruption, and DNA Extraction

Whole feces were collected from naturally infected humans and livestock animals; the samples contained *Cryptosporidium* oocysts morphologically indistinguishable from *C. parvum* by light microscopy (8,14). One sample of feces from a sheep experimentally infected with a standard (Moredun) strain originally of cervine origin (15) was included. Human feces were also tested in which no *Cryptosporidium* was detected but *Cyclospora* oocysts or *Giardia* cysts were detected by conventional techniques. All specimens were stored at 4°C without preservatives. Oocyst disruption and DNA purification were performed (8).

### Staining and Light Microscopy

Samples were reexamined by light microscopy after being stained by the modified Ziehl-Neelsen (MZN) acid-fast method (14) and the immunofluorescence (IF) method (8) with an anti-*Cryptosporidium*-oocyst monoclonal antibody designated MAB-C1 (16).

### PCR-RFLP analysis

PCR of two 18S rDNA fragments (reaction 1 [8,17] and reaction 2 [18]), COWP (5), TRAP-C1 (6), and TRAP-C2 (4) gene fragments, as well as restriction digestion with *Rsa*I for the COWP and TRAP-C1 genes, was performed. The 18S rDNA reaction 2 (18) was modified as described by Bornay-Llinares et al. (19) to include 4 mM $MgCl_2$, with a program cycle of 95°C for 5 min, 45 cycles of 94°C for 30 sec, 65°C for 30 sec, and 72°C for 1 min, followed by a final extension at 72°C for 9 min.

For the nested-COWP procedure (N-COWP), a 769-bp fragment of the COWP gene was amplified with primers BCOWPF (5'-ACCGCT TCTCAACAACCATCTTGTCCTC-3') and BCOWPR (5'-CGCACCTGTTCCCACTCAATGTA AACCC-3'), which encompasses the 553-bp fragment (5). Primers BCOWPNF and BCOWPNR were designed by using the PRIME program in the Genetics Computer Group Wisconsin package (Madison, WI). PCR amplification was performed in 25-µl volumes with 2.5 µl of DNA in 1x PCR buffer, 1.5 mM $MgCl_2$, 250 µM of each dNTP, 10 pmoles of each primer, and 1.25 units of Taq DNA polymerase. Samples were subjected to 30 cycles of 94°C for 1 min, 65°C for 1 min, and 72°C for 1 min, followed by a final extension at 72°C for 10 min. This single reaction is referred to as the extended-COWP (E-COWP) reaction. For the N-COWP procedure, a 553-bp gene fragment was then amplified from 2.5 µl of the E-COWP material as described (5), except that each primer (Cry9 and Cry15) was used at 10 pmoles. Positive and negative controls for all PCR procedures were included at all stages and for all batches.

For the N-COWP, E-COWP, COWP, 18S rDNA 1, 18S rDNA 2, TRAP-C1, and TRAP-C2 gene fragments, 5-µl aliquots of the PCR products were analyzed by electrophoresis in 1% agarose-ethidium bromide gels. *Rsa*I digestion of N-COWP, COWP, and TRAP-C1 fragments was resolved by electrophoresis in 3.2% typing-grade agarose gels containing ethidium bromide. All gels were recorded by using UV transillumination and Polaroid Type 667 film.

### DNA Sequencing

PCR products were purified in Microspin S-400 HR (Pharmacia Biotech, St. Albans, UK) and cloned by using the TOPO-TA Cloning kit (Invitrogen, Groningen, the Netherlands). Plasmid DNA was purified by using the Promega Wizard SV kit (Promega, Madison, WI), and clones were sequenced on an ABI377 automated sequencer with BigDye terminator chemistry with the M13(-21) primer at the Single Reaction DNA Sequencing Service (Cambridge Bioscience Ltd., Cambridge, UK). Sequences were analyzed with the Genetics Computer Group (GCG) program package (University of Wisconsin, Madison, WI).

### Results

### Nested COWP Procedure

Analysis of the published genotype 2 COWP gene sequence (GenBank accession numbers Z22537) led to design of two primers (BCOWPF and BCOWPR) to amplify a predicted E-COWP 769-bp fragment, which includes the 553-bp fragment amplified by the previously described Cry15/Cry9 primers (5). Appropriately sized fragments were generated by using the BCOWPF and BCOWPR primer pair from DNA extracted from human fecal samples containing *Cryptosporidium* genotypes 1, 2, and 3. The three respective amplicons were cloned and sequenced; the sequences are available from GenBank:

accession numbers af248741 (genotype 1), af248743 (genotype 2), and af248742 (genotype 3). Identical sequences were obtained from the genotype 2 sequence (accession number Z22537), with the exception of the insertion of three nucleotides.

The N-COWP amplification procedure for the 553-bp COWP fragment from the 769-bp E-COWP amplicon was developed and initially assessed by using 76 DNA samples extracted from whole human feces, which had been genotyped by using the COWP reaction. Identical results were obtained by the COWP and N-COWP procedures: 28 were genotype 1, 34 genotype 2, and 5 genotype 3; both genotypes 1 and 2 were recovered from nine samples. No amplicons were detected by the N-COWP procedure with DNA extracted from *Toxoplasma gondii* tachyzoites (two samples), *Eimeria tenella* oocysts (two samples), and feces containing either *Cyclospora* oocysts (10 samples) or *Giardia* cysts (11 samples).

### N-COWP by Different PCR Procedures

To assess the sensitivity of PCR procedures for the N-COWP reaction, amplification of DNA was compared with that from the E-COWP, COWP, TRAP-C1, and TRAP-C2, as well as the two 18S rDNA reactions. DNA samples prepared from whole feces were decimally diluted in water to $10^{-4}$, and each dilution was tested by all procedures. Samples were prepared from human feces containing genotypes 1, 2, or 3 and from ovine feces (Moredun strain) containing genotype 2 (Table 1).

The N-COWP reaction strongly amplified DNA from all samples to a dilution of $10^{-3}$, with the exception of the genotype 3 sample, in which

Table 1. Sensitivities of different polymerase chain reaction procedures for *Cryptosporidium* gene sequences

| Dilutions of | PCR procedure[a] | | | | | | |
|---|---|---|---|---|---|---|---|
| DNA sample | N-COWP | E-COWP | COWP | TRAP-C1 | TRAP-C2 | 18S rDNA 1 | 18s rDNA 2 |
| Human, genotype 1 | | | | | | | |
| Undiluted | +++[b] | ++ | ++ | ++ | ++ | ++ | ++ |
| $10^{-1}$ | +++ | ++ | ++ | ++ | ++ | ++ | ++ |
| $10^{-2}$ | +++ | + | ++ | — | + | + | ++ |
| $10^{-3}$ | +++ | — | — | — | — | + | ++ |
| $10^{-4}$ | — | — | — | — | — | — | — |
| Human, genotype 2 | | | | | | | |
| Undiluted | +++ | ++ | ++ | ++ | ++ | ++ | ++ |
| $10^{-1}$ | +++ | + | ++ | + | — | ++ | ++ |
| $10^{-2}$ | +++ | — | + | + | — | + | ++ |
| $10^{-3}$ | +++ | — | — | — | — | ± | ± |
| $10^{-4}$ | — | — | — | — | — | — | — |
| Human, genotype 3 (*Cryptosporidium meleagridis*) | | | | | | | |
| Undiluted | +++ | + | + | + | — | + | ++ |
| $10^{-1}$ | +++ | ± | — | — | — | + | ± |
| $10^{-2}$ | +++ | — | — | — | — | — | — |
| $10^{-3}$ | — | — | — | — | — | — | — |
| $10^{-4}$ | — | — | — | — | — | — | — |
| Ovine, genotype 2 (Moredun strain) | | | | | | | |
| Undiluted | +++ | ++ | ++ | ++ | ++ | ++ | ++ |
| $10^{-1}$ | +++ | + | ++ | + | + | ++ | ++ |
| $10^{-2}$ | +++ | ± | ± | — | — | + | ++ |
| $10^{-3}$ | +++ | — | — | — | — | — | ± |
| $10^{-4}$ | — | — | — | — | — | — | — |

[a]Polymerase chain reaction (PCR) procedures used for gene fragments: N-COWP = nested *Cryptosporidium* oocyst wall protein gene (553 bp, this study); E-COWP = extended COWP (769 bp, this study); COWP = unnested procedure (553 bp, 5); TRAP-C1 = thrombospondin-related adhesive protein *Cryptosporidium* 1 (506 bp, 6); TRAP-C2 = thrombospondin-related adhesive protein *Cryptosporidium* 2 (369 bp, 4); 18S rDNA 1 (422 bp, 8,17); 18S rDNA 2 (435 bp, 18).
[b]Semiquantitative results on the basis of strength of ethidium bromide staining in electrophoresis gels: strong (+++), moderate (++), weak (+), very weak (±), and amplification not detected(—).

amplification was achieved only up to $10^{-2}$ (Table 1). The two 18S rDNA procedures moderately amplified DNA to $10^{-3}$, with the exception of genotype 3, which gave only a weak reaction in the undiluted sample. The three PCRs used for genotyping (COWP, TRAP-C1, and TRAP-C2) all gave weaker signals than the N-COWP procedure, and product was sufficient for restriction enzyme digestion undiluted or at $10^{-1}$, except for genotype 3, in which there was insufficient amplification with all reactions, and COWP, in which there was sufficient amplification from the sample containing genotype 1 to perform genotyping at the $10^{-2}$ dilution (Table 1).

### Assessment of N-COWP Procedure

DNA was extracted from 1,680 fecal samples in which hospital laboratories had reported detection of *Cryptosporidium* oocysts by conventional procedures; these samples were from patients with diarrhea diagnosed in England, Northern Ireland, or Scotland during 1998-99. All samples were tested by the unnested COWP procedure, and those in which no amplicons were detected were retested by N-COWP. Samples were reexamined by microscopy if no amplification was detected by either COWP and N-COWP (except for two samples for which there was insufficient material) and a selection of other samples: overall, 475 (28%) and 397 (24%) of samples were retested by IF and MZN, respectively. Amplification and genotyping were successful in 95.2% of the samples, 77.6% by COWP and 17.6% by N-COWP (Table 2). Of the 43 samples in which no oocysts were detected, all were negative by COWP, N-COWP, and 18S rDNA-1 PCR. DNA was amplified from two of the 43 microscopy-negative samples by the 18S rDNA-2 reaction. Five of these microscopy-negative samples did not amplify DNA when tested with TRAP-C1.

Of the 35 COWP- and N-COWP-negative samples in which oocysts were detected after reexamination (Table 2), DNA was amplified from 11 (31%) by either 18S rDNA amplifications: three and four samples by 18S rDNA reactions 1 and 2, respectively, and four samples by both 18S rDNA amplifications.

Of the 1,600 samples in which DNA was amplified by either COWP or N-COWP (Table 2), 731, 209, and 210 were also tested by 18S rDNA reaction 1, 18S rDNA reaction 2, and TRAP-C2, respectively. DNA was amplified from 627 (86%), 166 (79%), and 138 (66%) by 18S rDNA reaction 1, 18S rDNA reaction 2, and TRAP-C2, respectively. Identical genotyping results were obtained by COWP/N-COWP and *Rsa*1 digestion of the TRAP-C1 fragment in all 138 samples in which amplification of the latter DNA fragment was achieved: 55 were genotype 1 and 83 genotype 2. The proportions of genotype 1 and genotype 2 amplifications were similar by COWP or N-COWP; however, there was a greater than tenfold increase in the proportions of both mixed genotypes 1 and 2 and genotype 3 detection by N-COWP (Table 2).

### COWP and N-COWP and Epidemiologic Studies

The COWP gene was amplified from 2,128 cryptosporidiosis cases: 71 from livestock animals and 2,057 from humans (Table 3). Among the samples from humans, a genotype was established by N-COWP but not by COWP in 476 (23.1%) of 2,057, 253 (35.8%) of 706, 13 (25.5%) of 51, and 210 (16.2%) of 1,300 of all samples, and those collected from drinking waterborne

Table 2. *Cryptosporidium* oocyst wall protein (COWP) gene analysis of DNA extracted from 1,680 human fecal samples

| PCR amplication procedure | No. of samples (%) | COWP genotypes 1 | 2 | 1 & 2 | 3 |
|---|---|---|---|---|---|
| COWP gene fragment amplified | | | | | |
| Unnested | 1,304 (77.6) | 381 | 917 | 2 | 4 |
| Nested[a] | 296 (17.6) | 81 | 198 | 7 | 10 |
| COWP gene fragment not amplified[b] | | | | | |
| Oocysts detected by microscopy | 35 (2.1) | | | | |
| Microscopy not reconfirmed[c] | 2 (0.1) | | | | |
| Oocysts not reconfirmed by microscopy | 43 (2.6) | | | | |

[a]All samples previously negative by unnested procedure.
[b]By both unnested and nested procedures.
[c]Insufficient material available.
PCR = Polymerase chain reaction.

Table 3. *Cryptosporidium* oocyst wall protein (COWP) gene analysis of DNA from 2,057 humans and 71 livestock animals

| | COWP genotypes | | | | |
|---|---|---|---|---|---|
| | 1 | 2 | 1 & 2 | 3 | Reference |
| Humans | | | | | |
| 2,057 cases | 795 | 1,227 | 20 | 15 | This study |
| Drinking water-associated outbreaks | | | | | |
| 1 | 140 | 2 | 3 | 0 | 20,21 |
| 2 | 158 | 14 | 1 | 1 | 22 |
| 3 | 4 | 0 | 0 | 0 | 20 |
| 4 | 15 | 0 | 0 | 0 | 23 |
| 5 | 0 | 6 | 0 | 0 | 24 |
| 6 | 0 | 25 | 0 | 0 | 24 |
| 7 | 4 | 331 | 0 | 2 | 24 |
| Swimming pool-associated outbreaks | | | | | |
| 8 | 3 | 0 | 0 | 0 | 24 |
| 9 | 6 | 3 | 0 | 0 | 25 |
| 10 | 0 | 10 | 0 | 0 | 25 |
| 11 | 9 | 1 | 3 | 0 | 25 |
| 12 | 14 | 2 | 0 | 0 | 25 |
| Sporadic human cases | 442 | 833 | 13 | 12 | 8, this study |
| Livestock animals | | | | | |
| Calves, lambs | 0 | 71 | 0 | 0 | This study |

outbreaks, swimming-pool outbreaks, and sporadic cases, respectively. Of samples from livestock animals, 10 (14.1%) of 71 genotypes were established by N-COWP but not COWP. Genotype 1 was found in 38.6% of the human samples, genotype 2 in 59.6%, both genotype 1 and 2 were detected in 1.0%, and genotype 3 (*C. meleagridis*) in 0.7%.

Genotyping results were obtained from 706 patients infected during seven drinking water-associated outbreaks: genotype 1 was predominantly recovered from patients in outbreaks 1 to 4, and genotype 2 from most of the patients in outbreaks 5 to 7 (Table 3). Genotyping results were obtained from 51 patients during five swimming pool-associated outbreaks (Table 3). Two of these outbreaks (8 and 10) were due to a single genotype, and the remaining three (9, 11, and 12) involved both genotypes 1 and 2 (Table 3). Two samples from swimming pool outbreak 6, which were from a single patient, yielded genotype 1 at first and both genotypes 1 and 2 six days later. Of 1,300 sporadic cases, 34.0% were genotype 1, 64.1% genotype 2, 1% were both genotypes 1 and 2, and 9% were genotype 3 (*C. meleagridis*).

### Conclusions

Human cryptosporidiosis has multiple potential host reservoirs of infection and multiple routes of transmission (1,2). Molecular biologic methods have allowed identification of two major genotypes within *C. parvum* (the principal infectious agent for human cryptosporidiosis) with two transmission cycles. The application of genotyping techniques may therefore provide a better understanding of the epidemiology of cryptosporidiosis, including different routes of transmission.

Epidemiologic studies of cryptosporidiosis have incorporated results from genotyping *C. parvum* (4,26-29), although these have been applied to relatively few samples. For example, among the estimated 400,000 cases associated with the 1993 waterborne outbreak in Milwaukee (30), genotyping data are available for five patients (all genotype 1 [4,26]). However, *C. parvum* genotype 1 was implicated in outbreaks associated with drinking and food, as well as person-to-person transmission in a day-care center and attendance at a water park (4,26-29). *C. parvum* genotype 2 was also associated with waterborne outbreaks, contaminated apple juice, and contact with cows (4,26,27). To investigate the epidemiology of cryptosporidiosis, we have described simple methods for the extraction of cryptosporidium DNA from whole feces and applied genotyping techniques to several hundred samples (8,10). We applied these techniques, together with the development and application of a sensitive PCR protocol (N-COWP), to >2,000 samples. Our techniques are less labor intensive than other methods (11,26,27) and allow analysis of large numbers of samples: we estimate that 1,000 samples can be extracted and genotyped within 6 months by one scientist working full time.

The N-COWP genotyping protocol is more sensitive than three unnested procedures (COWP, TRAP-C1, and TRAP-C2) also used for genotyping. The higher copy number of the 18S rDNA genes means that PCR procedures for these are likely to be more sensitive than those for the COWP, TRAP-C1, and TRAP-C2 gene sequences, and our data are consistent with this observation: the 18S rDNA genes occur as five copies (31), but the COWP, TRAP-C1 and TRAP-C2 genes occur as single copies per genome (32). Nested procedures for a single copy gene (the dihydrofolate reductase gene) and 18S rDNA genes were most sensitive when 11 PCR techniques for genotyping of *Cryptosporidium* were compared, although these studies were performed on DNA extracted from four semipurified oocyst suspensions (33).

One of the 18S rDNA amplifications reported elsewhere for genotyping (33) also amplified DNA from different *Cryptosporidium* species. However, Sulaiman and colleagues (33) reported that a COWP gene can be amplified from *C. serpentis* and *C. muris* (although the PCR products were faint) and that these are distinct from *C. parvum*. *C. wrairi* (5) and *C. meleagridis* (34) can be distinguished by PCR-RFLP analysis. We also reported that a single base mismatch (T to C substitution) occurs in the Cry9 COWP primer annealing region in genotype 3 (*C. meleagridis*) (13), which may account for the increased efficiency in amplification with the N-COWP procedure, as well as the faint amplifications reported for *C. serpentis* and *C. muris* (33). We are investigating the use of our extraction and PCR protocols described for identification of *Cryptosporidium* species, especially in samples that did not amplify COWP sequences but did amplify cryptosporidium 18S rDNA and in which oocysts were detected by microscopy.

A diagnosis of cryptosporidiosis can be established by examination of stained fecal smears prepared either directly from feces or after concentration (flotation) procedures (14). Although symptomatic cryptosporidiosis in humans is generally associated with large numbers of oocysts in the feces, infections occur in which oocysts cannot be detected by microscopy (14,35). Our DNA extraction method is based on whole feces; therefore, target DNA may be derived not only from oocysts, but also from other stages in the life cycle of this parasite. However, as found in experiments seeding DNA into feces, oocysts are the most likely source of DNA and the estimated sensitivity of the N-COWP reaction is equivalent to <500 oocysts/g of feces (Pedraza-Díaz et al., unpub data). Future studies will examine specimens from patients with diarrhea due to *Cryptosporidium* (and other intestinal pathogens) to establish the true sensitivity of this method for patient samples without detectable oocysts. Failure to detect oocysts may result from degradation of both oocysts and DNA, although DNA has been isolated and successfully amplified from whole fecal samples stored at 4°C for >5 years (8).

The N-COWP procedure detected a higher proportion of samples containing both genotypes 1 and 2. Further DNA sequence-based analysis indicates that these are true dual infections, not infections due to recombination within *C. parvum* (Pedraza-Díaz et al., unpub data). The increased rate of mixed infections identified by the N-COWP procedure is consistent with our data suggesting that the two genotypes may occur in feces at differing concentrations (8). Previously undetectable mixtures of genotypes may also explain apparent genetic changes due to selective growth as a result of host specificity after passage through different animals (7,36).

In this large series of cryptosporidiosis cases, all samples from livestock yielded genotype 2, consistent with previous results (9). Of >2,000 samples from humans, 38.6% were due to genotype 1, 59.6% to genotype 2, both genotypes 1 and 2 were recovered from 1%, and 0.7% were due to genotype 3 (*C. meleagridis).* There are relatively few comparative data analyzing larger series from humans, although Sulaiman et al. (26) reported that of 50 human cases, 82% were due to genotype 1 and 18% to genotype 2. These results with respect to the proportions of the *C. parvum* genotypes 1 and 2 differed markedly from our data for the United Kingdom; however, further results showed marked seasonal and geographic differences (34).

Data are presented here on 709 patients infected during seven drinking-waterborne outbreaks (51% of the microbiologically confirmed cases). Four of the outbreaks were almost exclusively due to genotype 1 and three to genotype 2. Data from field epidemiologic observations (23,24) suggest that contamination of water by sheep feces was involved in the three outbreaks due to genotype 2. All outbreaks occurred in the spring when lambing (as well as outbreaks of cryptosporidiosis in sheep) occurs most commonly in the United Kingdom (37). In contrast, the likely source of *C. parvum* in the four drinking-waterborne outbreaks predominantly due to genotype 1 was by contamination with human sewage; these occurred throughout the year. Outbreaks 1 and 2 occurred after heavy rain (20-22), and untreated sewage overflowing from storm drains may be a contributing factor.

Among the five outbreaks associated with swimming pools, one was due to genotype 1, one to genotype 2, and the remaining three to both genotypes 1 and 2. Outbreaks in swimming pools may be associated with fecal contamination from a single infected person (especially in toddler pools), so that a single genotype is recovered from the patients. However, outbreaks may also be due to more general problems such as contamination with sewage, poor disinfection, or inadequate maintenance of filtration equipment (25).

Our data on 1,300 sporadic cases, as well as further epidemiologic analysis (34), indicate that patients reporting contact with animals or farms were almost all infected by genotype 2; the spring peak in cases was almost exclusively due to genotype 2; genotype 1 was significantly more common in patients infected during the late summer-autumn peak and in those with a history of foreign travel; and there were distinct geographic variations in the distribution of the genotypes.

In summary, we described methods for the analysis of *Cryptosporidium* genotypes and demonstrated their application to a large series of samples. These approaches, together with the development of more discriminatory typing methods (28), should increase understanding of the epidemiology of cryptosporidiosis. Methods of improved sensitivity, such as those described here, will also be invaluable in detection and genotyping of *Cryptosporidium* in environmental samples.

### Acknowledgment

We thank colleagues in clinical microbiology laboratories for the donation of specimens.

The work of Dr. Pedraza-Díaz is funded by Biomed Grant PL 962557 from the European Commission and is jointly supervised by the Public Health Laboratory Service and the Imperial College of Science and Technology and Medicine, London, United Kingdom.

Dr. Pedraza-Díaz is a research student in the Central Public Health Laboratory of the PHLS. Her work focuses on the development of molecular biological methods for the study of intestinal infectious diseases.

### References

1. Fayer R, Speer CA, Dubey JP. The general biology of *Cryptosporidium*. In: Fayer R, editor. *Cryptosporidium* and cryptosporidiosis. Boca Raton: CRC Press; 1997. p. 1-41.
2. Meinhardt PL, Casemore DP, Miller KB. Epidemiological aspects of human cryptosporidiosis and the role of waterborne transmission. Epidemiol Rev 1996;18:118-36.
3. Awad-el-Kariem FM, Robinson HA, Dyson DA, Evans D, Wright S, Fox MT, et al. Differentiation between human and animal strains of *Cryptosporidium parvum* using isoenzyme typing. Parasitology 1995;110:129-32.
4. Peng MM, Xiao L, Freeman AR, Arrowood MJ, Escalante AA, Weltman AC, et al. Genetic polymorphisms among *Cryptosporidium parvum* isolates: evidence of two distinct human transmission cycles. Emerg Infect Dis 1997;3:567-73.
5. Spano F, Putignani L, McLauchlin J, Casemore DP, Crisanti A. PCR-RFLP analysis of the *Cryptosporidium* oocyst wall protein (COWP) gene discriminates between *C. wrairi* and *C. parvum* isolates of human and animal origin. FEMS Microbiol Lett 1997;150:209-17.
6. Spano F, Putignani L, Guida S, Crisanti A. *Cryptosporidium parvum*: PCR-RFLP analysis of the TRAP-C1 (thrombospondin-related adhesive protein of *Cryptosporidium*-1) gene discriminates between two alleles differentially associated with parasite isolates of animal and human origin. Exp Parasitol 1998;90:195-8.
7. Widmer G. Genetic heterogeneity and PCR detection of *Cryptosporidium parvum*. Adv Parasitol 1998;40:223-39.
8. McLauchlin J, Pedraza-Díaz S, Amar-Hoetzeneder C, Nichols GL. Genetic characterization of *Cryptosporidium* strains from 218 patients with diarrhea diagnosed as having sporadic cryptosporidiosis. J Clin Microbiol 1999;37:3153-8.
9. Morgan UM, Xiao L, Fayer R, Lal AA, Thompson RCA. Variation within *Cryptosporidium*: towards a taxonomic revision of the genus. Int J Parasitol 1999;29:1733-51.
10. Patel S, Pedraza-Díaz S, McLauchlin J, Casemore DP, Outbreak Control Team South and West Devon 1996, Incident Management Team and Further Epidemiological and Microbiological Studies Subgroup, North Thames, 1997. The molecular characterisation of *Cryptosporidium parvum* from two large suspected waterborne outbreaks. Commun Dis Public Health 1998;1:231-3.
11. Pieniazek NJ, Bornay-Llinares FJ, Slemenda SB, da Silva AJ, Moura INS, Arrowood M J, et al. New *Cryptosporidium* genotypes in HIV-infected persons. Emerg Infect Dis 1999;5:444-9.

12. Morgan UM, Weber R, Xiao L, Sulaiman I, Thompson RCA, Ndiritu W, et al. Molecular characterisation of *Cryptosporidium* isolates obtained from human immunodeficiency virus-infected individuals living in Switzerland, Kenya, and the United States. J Clin Microbiol 2000;38:1180-3.
13. Pedraza-Díaz S, Amar C, McLauchlin J. The identification and characterization of an unusual genotype of *Cryptosporidium* from human feces as *Cryptosporidium meleagridis*. FEMS Microbiol Lett 2000;189:189-94.
14. Arrowood MJ. Diagnosis. In: Fayer R, editor. *Cryptosporidium* and cryptosporidiosis. Boca Raton: CRC Press; 1997. p. 43-64.
15. Blewett DA. Quantitative techniques in *Cryptosporidium* research. In: Angus KW, Blewett DA, editors. Proceedings of the First International Workshop on Cryptosporidiosis. Edinburgh: The Animal Disease Research Association; 1989. p 85-95.
16. McLauchlin J, Casemore DP, Harrison TG, Gerson PJ, Samuel D, Taylor AG. The identification of *Cryptosporidium* oocysts by monoclonal antibody. Lancet 1987;i:51.
17. Patel S, Pedraza-Díaz S, McLauchlin J. The identification of *Cryptosporidium* species and *Cryptosporidium parvum* directly from whole feces by analysis of a multiplex PCR of the 18S rRNA gene and by PCR/RFLP of the *Cryptosporidium* outer wall protein (COWP) gene. Int J Parasitol 1999;29:1241-7.
18. Johnson DW, Pieniazek NJ, Griffin DW, Misener L, Rose JB. Development of a PCR protocol for sensitive detection of *Cryptosporidium* oocysts in water samples. Appl Environ Microbiol 1995;61:3849-55.
19. Bornay-Llinares FJ, da Silva AJ, Moura INS, Myjak P, Pietkiewicz H, Kruminis-Lozowska W, et al. Identification of *Cryptosporidium felis* in a cow by morphologic and molecular methods. Appl Environ Microbiol 1999;65:1455-8.
20. Bouchier I. *Cryptosporidium* in water supplies: Third report of the group of experts. London: Her Majesty's Stationery Office; 1998.
21. McLauchlin J, Casemore DP, Moran S, Patel S. The epidemiology of cryptosporidiosis: application of experimental sub-typing and antibody detection systems to the investigation of water-borne outbreaks. Folia Parasitol 1998;45:83-92.
22. Willocks L, Crampin A, Milne L, Seng C, Susman M, Gair R, et al. A large outbreak of cryptosporidiosis associated with a public water supply from a deep chalk borehole. Commun Dis Public Health 1998;1:239-43.
23. Surveillance of waterborne disease and water quality: January to June 1998. Commun Dis Public Health 1998;8:305-6.
24. Surveillance of waterborne disease and water quality. January to June 1999, and summary 1999. Commun Dis Public Health 1999;9:305-7.
25. Surveillance of waterborne disease and water quality: July to December 1999. Commun Dis Public Health 2000;10:65-7.
26. Sulaiman IM, Xiao L, Yang C, Escalante L, Moore A, Beard CB, et al. Differentiating human and animal isolates of *Cryptosporidium parvum*. Emerg Infect Dis 1998;4:681-5.
27. Ong CSL, Eisler DL, Goh SH, Tomblin J, Awad-el-Kariem FM, Beard CB, et al. Molecular epidemiology of cryptosporidiosis outbreaks and transmission in British Columbia, Canada. Am J Trop Med Hyg 1999;61:63-9.
28. Cacciò S, Homan W, Camilli R, Traldo G, Kortbeek T, Pozio E. A microsatellite marker reveals population heterogeneity within human and animal genotypes of *Cryptosporidium parvum*. Parasitology 2000;120:237-44.
29. Quiroz ES, Bern C, MacArthur JR, Xiao L, Fletcher M, Arrowood MJ, et al. An outbreak of cryptosporidiosis linked to a food handler. J Infect Dis 2000;181:695-700.
30. MacKenzie WR, Hoxie NJ, Proctor ME, Gradus MS, Blair KA, Peterson DE, et al. A massive outbreak in Milwaukee of *Cryptosporidium* infection transmitted through the public water supply. N Engl J Med 1994;331:161-7.
31. Le Blancq SM, Khramtsov NV, Zamani F, Upton SJ, Wun TW. Ribosomal RNA gene organization in *Cryptosporidium parvum*. Mol Biochem Parasitol 1997;90:463-78.
32. Piper M, Bankier AT, Dear PH. A HAPPY map of *Cryptosporidium parvum*. Genome Res 1999;8:1299-1307.
33. Sulaiman IM, Xiao L, Lal AA. Evaluation of *Cryptosporidium* genotyping techniques. Appl Environ Microbiol 1999;65:4431-35.
34. McLauchlin J, Amar C, Pedraza-Díaz S, Nichols GL. Molecular epidemiological analysis of *Cryptosporidium* spp. in the United Kingdom: results of genotyping *Cryptosporidium* spp. in 1,705 fecal samples from humans and 105 fecal from livestock animals. J Clin Microbiol 2000;38:3984-90.
35. Okhuysen PC, Chappell CL, Crabb JH, Sterling CR, DuPont HL. Virulence of three distinct *Cryptosporidium parvum* isolates for healthy adults. J Infect Dis 1999;180:1275-81.
36. Carraway M, Tzipori S, Widmer G. A new restriction fragment length polymorphism from *Cryptosporidium parvum* identifies genetic heterogeneous parasite populations and genetic changes following transmission from bovine to human hosts. Infect Immun 1997;65:3958-60.
37. Casemore DP. Epidemiological aspects of human cryptosporidiosis. Epidemiol Infect 1990;104:1-28.
